# Evaluation of the Safety of Percutaneous Sensory Nerve Stimulation in Patients with Head and Neck Cancer Receiving Chemoradiotherapy

**DOI:** 10.3390/jpm13071129

**Published:** 2023-07-12

**Authors:** Takao Hamamoto, Yuki Sato, Kohei Yumii, Nobuyuki Chikuie, Takayuki Taruya, Yuichiro Horibe, Takashi Ishino, Tsutomu Ueda, Sachio Takeno, Kenichi Yoshimura

**Affiliations:** 1 Department of Otorhinolaryngology, Head and Neck Surgery, Hiroshima University Hospital, 1-2-3, Kasumi, Minami-ku, Hiroshima 734-8551, Japan; takao0320@hiroshima-u.ac.jp (T.H.); sato0123@hiroshima-u.ac.jp (Y.S.); yumiik@hiroshima-u.ac.jp (K.Y.); housejak@hiroshima-u.ac.jp (N.C.); ttaruya@hiroshima-u.ac.jp (T.T.); horibey@hiroshima-u.ac.jp (Y.H.); tishino@hiroshima-u.ac.jp (T.I.); uedatsu@hiroshima-u.ac.jp (T.U.); 2 Medical Center for Translational and Clinical Research, Hiroshima University Hospital, Hiroshima 734-8555, Japan; keyoshim@hiroshima-u.ac.jp

**Keywords:** head and neck neoplasms, electric stimulation, chemoradiotherapy, swallowing, dysphagia

## Abstract

Chemoradiotherapy (CRT) is the standard treatment for locally advanced head and neck cancer; however, CRT may cause post-treatment dysphagia. Transcutaneous electrical sensory stimulation (TESS), developed in recent years for swallowing rehabilitation, is used at many medical facilities. Although TESS has been used for dysphagia in several fields, its safety and efficacy in patients with head and neck cancer remain to be clarified. Therefore, this study evaluated the safety of TESS in ten patients with head and neck cancers undergoing CRT. Swallowing rehabilitation intervention and TESS implementation were performed for all patients during CRT. Non-blood-toxicity adverse events (AEs), such as dermatitis and mucositis, occurred during CRT; however, the severity was less than grade 3. No patient experienced pain due to TESS. As survival time analysis using the Kaplan–Meier method for interferential current device implementation rates revealed a feasibility of 100% for up to 60 Gy and a feasibility of 78% for up to 70 Gy, TESS may be feasible until 70 Gy. This study confirmed the feasibility and safety of TESS in the head and neck region during CRT. Although the precise mechanism of TESS on dysphagia remains unclear, its continued use has great potential for improving sensory disturbance.

## 1. Introduction

Chemoradiotherapy (CRT) is currently the standard treatment for locally advanced head and neck cancer [1]. However, CRT can lead to the incidence of mucositis, dermatitis, muscle and nerve dysfunction, and tissue fibrosis, resulting in post-treatment dysphagia [2]. Early rehabilitation intervention is recommended for the prevention of dysphagia. Electrical stimulation has been recommended as another option [3]. The efficacy of neuromuscular electrical stimulation (NMES) in treating dysphagia after CRT has been demonstrated in several studies. In recent years, transcutaneous electrical sensory stimulation (TESS), which is similar to NMES, has been developed for treating dysphagia. Interference-wave electrical stimulation (IFES) is used for performing TESS, whereas pulsed-wave electrical stimulation is used for performing NMES. IFES stimulates deep tissues with two electrical stimuli that have slightly different frequencies, creating undulations through local wave interference. In general, TESS has been known to result in lesser irritation to the skin than NMES. Clinical trials evaluating the use of TESS for dysphagia have reported its efficacy in patients with various types of dysphagia, including sequelae of cerebrovascular disorders [3,4,5]. Based on this evidence, an interferential current device (IFCD) was developed in Japan and received medical device certification under the trade name “Gentle-stim” in July 2015. It is now commercially available and is used at many medical facilities in Japan for swallowing rehabilitation. Although the use of TESS with an IFCD for dysphagia has been clinically studied in several fields, its safety and efficacy in patients with head and neck cancer have not yet been investigated.

The IFCD has been recognized as a noninvasive medical device, and its likelihood of resulting in the incidence of serious adverse events (AEs) is extremely low. However, the insert instructions for the IFCD recommend that it should be used with caution under a physician’s guidance in inpatient settings, not outpatient settings, as its safety has not been established in patients with malignant tumors or acute illnesses. Similarly, its use in acutely injured or inflamed areas should be avoided as its safety has not been established. Thus, due to these safety considerations, IFCDs are only approved for use under the supervision of a healthcare professional and are not recommended for use by the patient alone, such as at home.

In this study, the safety of TESS in patients with head and neck cancers undergoing CRT was evaluated. This study aimed to determine whether TESS with an IFCD exacerbates the disease status, pain, and feasibility of the CRT treatment. In addition, we reported the effectiveness of electrical stimulation therapy as rehabilitation therapy for dysphagia after CRT, along with a discussion of the literature.

## 2. Materials and Methods

### 2.1. Ethics

The study protocol was approved by the Certified Clinical Research Committee of Hiroshima University (certification number: CRB210005), registered with the Japan Registry of Clinical Trials (jRCTs062220008), and submitted to the Ministry of Health, Labor and Welfare. Written informed consent was obtained from each participant, and the study was conducted in accordance with the tenets of the Declaration of Helsinki.

### 2.2. Study Objectives and Eligibility Criteria

This single-center, exploratory, single-arm prospective study was conducted to evaluate the safety of TESS in patients enrolled and treated between 13 April 2022 and 30 March 2023. Ten patients with locally advanced head and neck cancer who underwent CRT were selected from the Hiroshima University Hospital. The eligibility criteria were as follows: (1) patients who underwent CRT for head and neck cancer at the Hiroshima University Hospital; (2) patients who received 70 Gy of radiation to the laryngopharyngeal area, including the nasopharynx, oropharynx, hypopharynx, or larynx; (3) patients over 20 years of age at the time of obtaining consent; and (4) patients who could provide written consent for participation in this study. The exclusion criteria were as follows: (1) patients with a history of receiving radiation therapy in the head and neck region, (2) patients with a history of undergoing a tracheostomy, (3) patients with a history of receiving radiation therapy mainly in an area other than the laryngopharyngeal area, (4) patients with pacemakers and implantable cardioverter-defibrillators, (5) patients with difficulty wearing IFCD on the neck, (6) patients with many inconveniences in daily life (performance status 2 or higher), (7) patients who were pregnant, could conceive, or were breastfeeding, and (8) patients who were judged to be inappropriate by the principal investigator or the research coordinator

### 2.3. Medical Device Used

**Figure 1:**
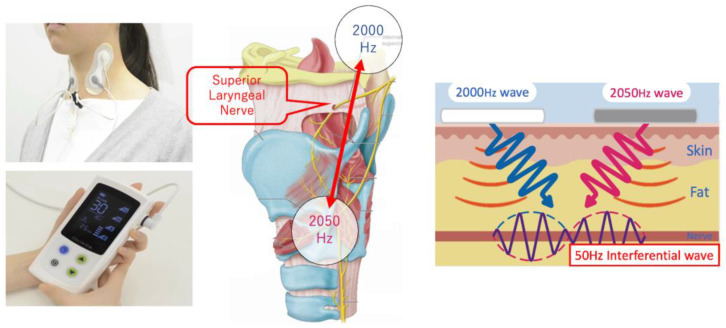
Interferential current device named “Gentle-Stim”.

We used an IFCD named “Gentle-Stim” (Food Care Co., Ltd., Sagamihara, Japan, https://www.food-care.co.jp/lng_en/message.html, accessed on 11 May 2023, medical device certification number: 227AHBZX00026000) for TESS in this study. The indications and effects of this device include percutaneous nerve and muscle stimulation for analgesia and amelioration of muscle atrophy (Figure 1).

Gentle-Stim is an inferential current device manufactured by the Food Care Corporation. The mechanism of this device consists of electrodes placed across the hyoid bone and thyroid cartilage that create interference waves, stimulate the superior laryngeal nerve, and improve laryngeal sensation.

### 2.4. Interventions

**Figure 2:**
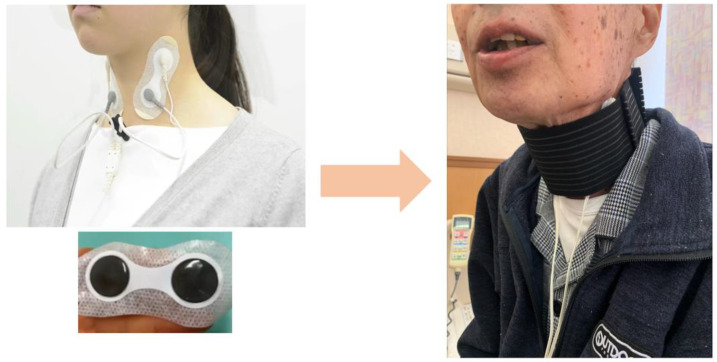
Procedures of the electrodes’ attachment.

The IFCD was placed during swallowing rehabilitation. After removing sebum, sweat, and dirt from the cervical skin, the stimulating electrodes were placed with a neck band, as adhesive tape can cause skin deprivation when removed (Figure 2). The stimulation duration was 30 min, and the stimulation output level was adjusted after assessing the patient. In cases where swallowing rehabilitation could not be performed owing to medical conditions, the IFCD placement without rehabilitation was allowed.

As adhesive sheets used for attaching the electrodes have a tendency to worsen dermatitis, a belt was wrapped around the neck during chemoradiotherapy.

### 2.5. Definition of “Feasible”

If the patient was able to wear the IFCD with a 10 Gy irradiation period of at least 3 out of 5 days and if it was used for at least 20 min at a power exceeding level 1, it was considered “feasible.” If the patient refused to wear the device or if the physician determined the treatment to be unsuitable, it was considered “not feasible.” The presence or absence of swallowing rehabilitation did not affect the use of the IFCD.

### 2.6. Outcome Measures

As irradiation progressed, AEs, such as dermatitis, mucositis, and pain, were expected to become more severe owing to cumulative toxicity. AEs were classified according to the Common Terminology Criteria for Adverse Events (version 5.0; translated into Japanese by the Japan Clinical Oncology Group) [6]. Even if the IFCD placement could be performed without complications in the early period, the IFCD placement was assumed to be difficult in the final stages. Therefore, we performed an evaluation at every 10 Gy irradiation dose and examined the feasibility rate and duration. In addition, the number of days of swallowing rehabilitation intervention, number of days of IFCD implementation, output power, intervention duration, and AEs were also evaluated.

### 2.7. Primary Endpoint and Statistical Analysis

The primary endpoint of this study was the rate of feasibility of the IFCD. A Kaplan–Meier survival-time analysis was performed using a time axis of 10 Gy units for the cumulative irradiation dose to account for termination. Treatment was terminated if the radiation therapy was discontinued for any reason. To evaluate the feasibility rate in units of 10 Gy, the cumulative irradiation dose used as the time axis was rounded off to the nearest 10 Gy. The feasibility rate of the IFCD was acceptable if it exceeded 70% at a 10 Gy unit cumulative irradiation dose. If more than 7 of 10 enrolled patients were feasible, the lower limit of the 90% one-sided confidence interval of the rate of feasibility of the IFCD would exceed 45%. A sample size of 10 patients was required to obtain a sufficient precision for this exploratory trial for safety. All statistical analyses were performed using JMP Pro ver.16.2.0 (SAS Institute Inc., Cary, NC, USA). 

## 3. Results

The 10 enrolled patients were all males, with an average age of 64.8 years. Among them, seven patients had hypopharyngeal cancer, one patient had nasopharyngeal cancer, one patient had laryngeal cancer, and one patient had unknown primary cancer. Two patients had stage II cancer, four patients had stage IVA cancer, and four patients had stage IVB cancer. Six patients received induction chemotherapy (docetaxel, cisplatin, and 5-fluorouracil) before CRT, four patients received two cycles of triweekly cisplatin, five patients received three cycles of triweekly cisplatin, and one patient received seven cycles of weekly cetuximab during irradiation. As for radiation completion, nine patients received an irradiation dose of 70 Gy, whereas the treatment was terminated after an irradiation dose of 66 Gy at the patient’s request (Table 1).

Swallowing rehabilitation intervention and IFCD implementation were performed on almost all days throughout CRT. On these days, the average output power of the IFCD ranged from level 7 to level 8, which was unrelated to the accumulated radiation dose (*p* = 0.7926). In addition, almost all patients underwent TESS for 30 min. Swallowing rehabilitation and IFCD were performed at doses of up to 64 Gy in one case, up to 66 Gy in three cases, up to 68 Gy in two cases, and up to 70 Gy in four cases. Patients who had difficulty implementing the IFCD at the end of treatment also had difficulty with the swallowing rehabilitation intervention (Table 2).

Although non-blood-toxicity AEs, such as dermatitis, mucositis, dry mouth, and abnormal taste, occurred during CRT, the severity was less than grade 3 (Table 3). None of the patients complained of pain caused by IFCD electrical stimulation. 

All patients were able to wear the IFCD for more than 3 out of 5 days for a duration of more than 20 min. In addition, the power exceeded level 1 until reaching an irradiation dose of 60 Gy. Therefore, the IFCD implementation was feasible for all patients until 60 Gy. On reaching an irradiation dose of 70 Gy, seven patients were able to accept the IFCD implementation for more than 3 days. However, two patients declined to wear the IFCD due to contact pain, and one patient terminated the radiation treatment after reaching a dose of 66 Gy due to general fatigue and pain. A survival-time analysis using the Kaplan–Meier method for IFCD implementation rates showed a feasibility of 100% for up to 60 Gy and a feasibility of 78% for up to 70 Gy (Table 4). The analysis for the primary endpoint revealed that the IFCD implementation was feasible until an irradiation dose of 70 Gy. 

## 4. Discussion

Surgery is the mainstay of treatment for early-stage head and neck cancer. In contrast, advanced head and neck cancers are treated with a combination of radiation therapy, chemotherapy, and surgery. Although radical surgery for advanced head and neck cancer is highly curative, it requires the simultaneous removal of the surrounding organs, muscles, vascular vessels, and nerves and may result in the permanent loss of speech, swallowing function, and changes in appearance. Pignon et al. reported on a large meta-analysis in 2000 and 2009 that demonstrated the efficacy of CRT, which is now recognized as the standard treatment for advanced head and neck cancer “for organ-sparing purposes” [7]. Although CRT can lead to the incidence of mucositis, dermatitis, and dysgeusia during treatment, tissue scarring, muscle atrophy, sensory loss, dysphagia, and aspiration are often observed [2]. In some cases of severe dysphagia, patients have difficulty with oral intake due to repeated aspiration pneumonia, even though “organs could be preserved”. In other words, “organ preservation” in CRT may not always lead to “function preservation” [8]. Therefore, in CRT, for the purpose of “organ preservation”, established methods to obtain “functional preservation” as well as cancer treatment have been explored. Induction chemotherapy with cisplatin and 5-fluorouracil with docetaxel may have benefit in terms of organ preservation and the reduction of local and distant failure rates in some populations [9,10]. However, the value for survival and functional preservation remains unclear despite decades of research [11,12].

The main causes of dysphagia after CRT are “weakness of the swallowing muscles” and “decreased sensation in the pharynx”. Evidence accumulated in recent years has shown that the use of various neuromuscular electrical stimulators in combination with general swallowing rehabilitation has an additive effect [3].

NMES is recognized as a rehabilitation technique for the weakness of the swallowing muscles. In a randomized controlled trial of patients with nasopharyngeal cancer after radiation therapy, Lin et al. reported that swallowing rehabilitation with NMES significantly improved the swallowing function [13]. Long et al. also reported a significant improvement in the swallowing angiography parameters in the NEMS plus balloon dilation group in a randomized controlled trial including the same participants [14]. These studies were performed during the post-treatment period with some concerns that its use during CRT may cause pain as NMES using “pulsed-wave electrical stimulation” is intended to produce passive muscle contraction. TESS is recognized as a rehabilitation method for the loss of sensation in the pharynx. TESS is an IFC-based treatment that stimulates deep tissues by applying two medium-frequency electrical stimuli of slightly different frequencies to a local area, creating undulations through wave interference. As it is a new treatment, there is no evidence of its efficacy in the head and neck region.

Although TESS is considered to cause lesser irritation to the skin than NMES and can be performed during CRT, it was necessary to confirm its safety. The timing of the therapeutic intervention was selected during inpatient treatment, which allowed for the most intensive therapeutic intervention. Safety was determined during CRT, which is the most severe situation. The IFCD was 100% feasible up to 60 Gy and 78% feasible up to 70 Gy, and the time required to perform IFCD was 30 min, which exceeded the prescribed duration of 20 min in almost all cases. Although the pain of dermatitis was most severe at the end of the treatment during the 70 Gy irradiation dose unit period, some patients refused to wear the IFCD. The pain was thought to be caused by contact rather than by the electrical stimulation itself. The range of cumulative irradiation doses that exceeded the 70% feasibility rate was defined as the feasible range. Thus, the IFCD was considered feasible until the end of the CRT treatment. Although the output power seemed to change in relation to the radiation dose accumulation, no significant correlation was observed. Alternative methods, such as fixing the electrodes with a band, have been used to avoid the risk of the adhesive tape causing cervical skin peeling. Additionally, the IFCD stimulation did not cause pain during the study period. All AEs that occurred during the treatment period had a severity of grade 3 or less, and no serious AEs related to the IFCD intervention were observed during the study period.

Several studies have explored the treatment of dysphagia in patients with head and neck cancer undergoing CRT. Kotz et al. conducted a randomized controlled study and reported that swallowing function after CRT was significantly better in the rehabilitation group than that in the no-rehabilitation group, as assessed by the Functional Oral Intake Scale [15]. Mann et al. conducted a randomized controlled trial for swallowing rehabilitation during CRT and reported that the rehabilitation group had significantly better results [16]. Tang et al. also conducted a randomized controlled trial of patients with nasopharyngeal cancer and reported that the post-treatment rehabilitation group had a significantly better ability to swallow than the no-treatment group, as assessed by a water drinking test [17]. Kulbersh et al. studied the adequate timing of swallowing rehabilitation and reported that the quality-of-life scores related to dysphagia were significantly higher in the group that received prophylactic swallowing rehabilitation than those in the group that received rehabilitation after treatment [18].

In advanced head and neck cancer, it is not always possible to perform rehabilitation intervention before treatment as some patients experience swelling, pain on swallowing, and dyspnea. Prophylactic rehabilitation, similar to post-treatment rehabilitation, largely depends on the patient’s proactivity, and rehabilitation in outpatient clinics has limitations in terms of duration and means. Therefore, patients must continue their rehabilitation after discharge from the hospital. In Japan, TESS is recommended to be used under the guidance of a medical healthcare provider, and self-administration by patients is not considered to be safe [19]. The findings of the present study demonstrate that TESS could be performed safely during CRT, thereby supporting the expansion of the range of indications for TESS. A limitation of this study is that the results do not guarantee the safety of TESS for all patients in all situations. It should be noted that the range of irradiation and dose of radiation to the neck differ depending on the primary lesion and metastatic neck lymph nodes. However, the cases comprising the study population in this study included a wide variety of participants. Although the irradiation dose of the primary unknown cancer did not reach the definitive dose required in the laryngopharyngeal area, the irradiated field covered the whole neck. The participants included in this study were receiving concurrent chemoradiotherapy as well as chemoradiotherapy followed by induction chemotherapy. It has been reported that even patients receiving bioradiotherapy who developed severe mucositis and dermatitis could complete TESS intervention until the end of the treatment period [20], indicating that TESS is feasible regardless of the primary lesions, extent of irradiation, radiation doses, and combined chemotherapy agents. All patients included in this study participated enthusiastically in the rehabilitation process; however, this situation does not reflect the real-world scenario. Lastly, another limitation of this study would be the selection bias. As CRT may cause severe dermatitis and mucositis, the use of TESS continues to require medical supervision and careful follow-up during CRT. Thus, TESS can be used safely with prudent measures.

Intensive swallowing rehabilitation interventions should be preferably performed during hospitalization for CRT and after radical treatment. Although the effect of TESS on dysphagia is not yet clear, its continued use by patients themselves at home has great potential to contribute to the improvement of sensory disturbance. As TESS is an easy, passive, and harmless rehabilitation technique, we suggest that the restrictions surrounding its use be lifted to enable its use by outpatients after discharge. In order to examine its safety and effectiveness on swallowing function after CRT, the patients treated with TESS will continue to be followed up. Based on its safety in patients with head and neck cancer after CRT, we aim to assess the improvement in swallowing function before and after treatment in patients with dysphagia after CRT. Nevertheless, the effectiveness of TESS must be evaluated by further studies in the future.

## 5. Conclusions

This study confirmed the feasibility and safety of TESS with IFCD in the head and neck region, even during CRT. Although the effectiveness of this method needs to be further investigated in more cases, TESS may be another treatment option for hyposensitivity after CRT.

## Figures and Tables

**Table 1:** Patients’ background.

No.	Age/Sex	Primary Site	TNM/Stage	Treatment	Chemo Agent/Cycles
1	70/Male	Hypopharynx	T1N2bM0/stage IVA	CRT after IC 2 cycles	Cisplatin/3 cycles
2	59/Male	Nasopharynx	T1N1M0/stage II	CRT after IC 2 cycles	Cisplatin/2 cycles
3	73/Male	Primary unknown	T0N3bM0/stage IVB	CRT	Cisplatin/3 cycles
4	76/Male	Hypopharynx	T4aN2bM0/stage IVA	BRT	Cetuximab/7 cycles
5	72/Male	Hypopharynx	T2N3bM0/stage IVB	CRT after IC 2 cycles	Cisplatin/3 cycles
6	64/Male	Hypopharynx	T4aN2cM0/stage IVA	CRT after IC 2 cycles	Cisplatin/2 cycles
7	45/Male	Hypopharynx	T4bN3bM0/stage IVB	CRT after IC 2 cycles	Cisplatin/2 cycles
8	74/Male	Hypopharynx	T4aN3bM0/stage IVB	CRT after IC 2 cycles	Cisplatin/2 cycles
9	51/Male	Larynx	T2N0M0/stage II	CRT	Cisplatin/3 cycles
10	64/Male	Hypopharynx	T1N2bM0/stage IVA	CRT	Cisplatin/3 cycles

CRT, chemoradiotherapy; IC, induction chemotherapy; BRT, bioradiotherapy.

**Table 2:** Days of swallowing rehabilitation and interferential current device (IFCD) implementation, IFCD output power and procedure time at every 10 Gy stage, and intervention period.

No.	Days of SR	Days of IFCD	Output Power	Procedure Time	IP/TID
1	5-5-5-4-5-5-4	4-5-5-5-5-5-4	13-13-12-12-12-12-13	29-30-30-30-30-30-30	68 Gy/70 Gy
2	5-5-5-5-5-5-2	4-5-5-5-5-5-2	9-5-6-6-11-6-7	30-30-30-30-30-30-30	64 Gy/70 Gy
3	5-5-5-5-5-5-5	5-5-5-5-5-5-5	8-7-5-7-7-10-13	30-30-30-30-30-30-30	66 Gy/66 Gy
4	4-5-5-5-5-5-5	4-5-5-5-5-5-1	3-7-7-3-4-1-1	30-30-30-30-30-30-30	62 Gy/70 Gy
5	4-5-5-5-5-5-5	4-5-5-5-5-5-5	9-6-9-8-8-9-8	30-30-30-30-30-30-30	70 Gy/70 Gy
6	5-5-4-5-4-5-3	5-5-4-5-4-5-3	13-13-15-12-10-10-10	30-30-30-30-30-30-30	66 Gy/70 Gy
7	4-5-5-5-4-5-5	4-5-5-5-5-5-5	5-4-4-7-3-2-3	30-30-30-30-30-30-30	70 Gy/70 Gy
8	4-5-5-5-5-5-5	4-5-5-5-5-5-5	5-3-2-2-2-1-1	30-30-30-30-30-30-30	70 Gy/70 Gy
9	4-5-3-5-5-5-5	4-5-3-5-5-5-5	5-7-8-8-10-11-11	30-30-30-30-30-30-30	66 Gy/70 Gy
10	4-5-3-4-5-5-5	4-5-3-4-5-5-5	6-7-8-6-8-8-7	30-30-30-30-30-30-30	68 Gy/70 Gy
	*p* = 0.8493	*p* = 0.8767	*p* = 0.7926	*p* = 0.1347	

SR, swallowing rehabilitation; IFCD, interferential current device; IP, intervention period; TID, total irradiation dose.

**Table 3:** Adverse events (nonhematological toxicities).

Adverse Event	Grade 1	Grade 2	Grade 3	Grade 4	Grade 5
Dermatitis	3	4	3	0	0
Mucositis	4	4	2	0	0
Dry mouth	4	5	1	0	0
Dysgeusia	3	5	0	0	0
Aspiration	1	0	0	0	0
Alopecia	1	0	0	0	0

(The table shows the number of patients and types of adverse events).

**Table 4:** Primary endpoint, the rate of feasibility of the interferential current device (IFCD).

No.	10 Gy	20 Gy	30 Gy	40 Gy	50 Gy	60 Gy	70 Gy
1	F	F	F	F	F	F	F
2	F	F	F	F	F	F	NF
3	F	F	F	F	F	F	- *
4	F	F	F	F	F	F	NF
5	F	F	F	F	F	F	F
6	F	F	F	F	F	F	F
7	F	F	F	F	F	F	F
8	F	F	F	F	F	F	F
9	F	F	F	F	F	F	F
10	F	F	F	F	F	F	F
RF **	100%	100%	100%	100%	100%	100%	78%

F, feasible; NF, not feasible; RF, the rate. * terminated after 66 Gy, ** survival time analyzed using the Kaplan–Meier method.

## Data Availability

The data presented in this study are available upon reasonable request from the corresponding author.
